# Feasibility and acceptability of transcranial stimulation in obsessive–compulsive symptoms (FEATSOCS): study protocol for a randomised controlled trial of transcranial direct current stimulation (tDCS) in obsessive–compulsive disorder (OCD)

**DOI:** 10.1186/s40814-021-00945-6

**Published:** 2021-12-06

**Authors:** Eduardo Cinosi, David Adam, Ibrahim Aslan, David Baldwin, Kieran Chillingsworth, Arun Enara, Tim Gale, Kabir Garg, Matthew Garner, Robert Gordon, Natalie Hall, Nathan T. M. Huneke, Sonay Kucukterzi-Ali, Joanne McCarthy, Daniel Meron, Deela Monji-Patel, Roisin Mooney, Trevor Robbins, Megan Smith, Nick Sireau, David Wellsted, Solange Wyatt, Naomi A. Fineberg

**Affiliations:** 1 Highly Specialised OCD and BDD Service, Hertfordshire Partnership NHS University Foundation Trust, Rosanne House, Parkway, Welwyn Garden City, Hertfordshire UK; 2 University of Hertfordshire, Hertfordshire, UK; 3 ORCHARD-Advancing Global OCD Research Charity, Cambridge, UK; 4 Faculty of Medicine, Clinical and Experimental Sciences (CNS and Psychiatry), University of Southampton, Southampton, UK; 5 Southern Health NHS Foundation Trust, Southampton, UK; 6 Somerset NHS Foundation Trust, Taunton, UK; 7 Department of Psychology, Behavioural and Clinical Neuroscience Institute, University of Cambridge, Cambridge, UK

**Keywords:** Obsessive–compulsive disorder (OCD), Transcranial direct current stimulation (tDCS), Noninvasive neurostimulation, Feasibility study, Randomised controlled trial

## Abstract

**Background:**

Obsessive–compulsive disorder (OCD) is a neuropsychiatric disorder which often proves refractory to current treatment approaches. Transcranial direct current stimulation (tDCS), a noninvasive form of neurostimulation, with potential for development as a self-administered intervention, has shown potential as a safe and efficacious treatment for OCD in a small number of trials. The two most promising stimulation sites are located above the orbitofrontal cortex (OFC) and the supplementary motor area (SMA).

**Methods:**

The aim of this feasibility study is to inform the development of a definitive trial, focussing on the acceptability, safety of the intervention, feasibility of recruitment, adherence and tolerability to tDCS and study assessments and the size of the treatment effect. To this end, we will deliver a double-blind, sham-controlled, crossover randomised multicentre study in 25 adults with OCD. Each participant will receive three courses of tDCS (SMA, OFC and sham), randomly allocated and given in counterbalanced order. Each course comprises four 20-min stimulations, delivered over two consecutive days, separated by at least 4 weeks’ washout period. We will collect information about recruitment, study conduct and tDCS delivery. Blinded raters will assess clinical outcomes before, during and up to 4 weeks after stimulation using validated scales. We will include relevant objective neurocognitive tasks, testing cognitive flexibility, motor disinhibition, cooperation and habit learning.

**Discussion:**

We will analyse the magnitude of the effect of the interventions on OCD symptoms alongside the standard deviation of the outcome measure, to estimate effect size and determine the optimal stimulation target. We will also measure the duration of the effect of stimulation, to provide information on spacing treatments efficiently. We will evaluate the usefulness and limitations of specific neurocognitive tests to determine a definitive test battery. Additionally, qualitative data will be collected from participants to better understand their experience of taking part in a tDCS intervention, as well as the impact on their overall quality of life. These clinical outcomes will enable the project team to further refine the methodology to ensure optimal efficiency in terms of both delivering and assessing the treatment in a full-scale trial.

**Trial registration:**

ISRCTN17937049. (date applied 08/07/2019).

Recruitment (ongoing) began 23rd July 2019 and is anticipated to complete 30th April 2021.

## Background

With a lifetime prevalence of 2–3%, obsessive–compulsive disorder (OCD) represents a leading global cause of functional disability [1, 2]. Affecting all population groups, regardless of gender and culture, OCD typically has an early onset, follows a prolonged course, and is associated with significant reduced quality of life (QoL) and social and occupational impairment [3]. Chronic OCD is associated with substantial comorbidity and considerable disability. The damage to psychosocial function is comparable to schizophrenia [3]. Severe OCD is associated with long-term hospitalisation, residential care and suicidal behaviour [4–8]. The economic impact of OCD to the individual, family and society is considerable in terms of direct and indirect costs [9]. Whereas evidence-based treatments lead to symptomatic improvement and associated improved QoL [10], rates of incomplete recovery and treatment resistance are high: approximately 40% patients do not respond, and 50% need further treatment [4]. Delayed treatment prolongs illness and reduces therapeutic gain [11]. New treatments are needed to improve health outcomes [12, 13].

Brain imaging demonstrates abnormal cortico-striatal neurocircuitry as underlying OCD pathology [14–17]. Studies show that targeting this circuitry with ablative neurosurgery and invasive neurostimulation with deep brain stimulation (DBS) improves OCD, possibly by enhancing information processing [4, 15, 18]. In 2009, DBS received the Food and Drug Administration (FDA) humanitarian use approval and the Conformité Européenne (CE) mark for the treatment of refractory OCD, making this disorder the first psychiatric indication approved for DBS treatment [19]. On the other hand, DBS is invasive, costly and burdensome and reserved for patients with severe, refractory OCD [15]. Noninvasive neurostimulation of superficial cortical nodes is safer and may potentially be applied earlier in the course of illness. Evidence from randomised controlled trials supports the efficacy of repetitive transcranial magnetic stimulation (rTMS) in OCD: meta-analyses have identified the orbitofrontal cortex (OFC) and supplementary motor area (SMA) as the most promising targets [20, 21]. In 2014, deep transcranial magnetic stimulation (d-TMS) was the first noninvasive neuromodulation treatment receiving EU marketing approval for the treatment of resistant OCD. However, TMS is also relatively costly, involves technical equipment and cannot be delivered in patients’ homes.

Transcranial direct current stimulation (tDCS) is an alternative noninvasive neurostimulation tool which involves administration of low-amplitude (1–2 mA) electric current to the brain between a cathode and anode. Anodal tDCS is thought to enhance cortical excitability and cathodal tDCS to have an inhibitory effect [22]. TDCS may be a preferable option as it is cheaper, portable, simple and safe to use [23]. Systematic reviews and meta-analyses indicate adequate safety, tolerability and potential efficacy in depression and other psychiatric disorders including OCD [22, 24–30]. The National Institute of Clinical Excellence (NICE) identified that the evidence on tDCS for depression raises no major safety concerns and encourage further research into tDCS [31].

Research into tDCS in OCD is still in its infancy. Preliminary evidence from small uncontrolled studies and case reports in treatment-resistant OCD patients confirm its safety and suggest encouraging efficacy results using protocols targeting OFC, SMA and other cortical regions (such as dorsolateral prefrontal cortex and dorsomedial prefrontal cortex) [22, 26–29, 32–34]. Studies present significant heterogeneity and methodological differences in sample selection criteria, concomitant treatment and tDCS stimulation protocols [22, 29, 33]. A randomised sham-controlled trial (*n* = 24 treatment-resistant OCD subjects) demonstrated efficacy for anodal tDCS administered over bilateral pre-SMA and cathodal tDCS over right supra-orbital regions [35]. However, a previous randomised crossover trial (*n* = 12) found clinical improvement with cathodal tDCS over pre-SMA, while anodal tDCS was ineffective [36]. Thus, replication studies are needed to determine the optimal stimulation protocol for tDCS over SMA in OCD. Another randomised, sham-controlled trial (*n* = 21 treatment-resistant OCD patients) using cathodal tDCS delivered over the OFC and the anode over the right cerebellum showed significant acute reduction of OCD symptoms immediately after the tDCS regimen compared with sham stimulation [37]. However, active tDCS was not superior to sham stimulation in alleviating OCD symptoms at the follow-up (12-week period) [37].

A recent double-blind, randomised, and sham-controlled study investigated the efficacy of tDCS as add-on treatment for treatment-resistant OCD (*n* = 43) [38]. Over 20 consecutive weekdays of active or sham tDCS sessions (30 min, cathode was positioned over the SMA and the anode over the left deltoid) were administered, followed by an 8-week follow-up. Patients that received active tDCS achieved a significant reduction of OCD symptoms than those that received sham at week 12, with mean (*SD*) Yale-Brown Obsessive–Compulsive Scale (Y-BOCS) score changes of 6.68 (5.83) and 2.84 (6.3) points, respectively (Cohen’s *d*: 0.62 (0.06–1.18), *p* = 0.03), with no between-group differences in responders (four patients in the active tDCS and one in the sham group) [38]. There were no significant effects in reducing symptoms of depression or anxiety, and patients in both groups reported mild adverse events [38]. Also based on theoretical and computational models, authors suggest that overall cathodal tDCS may be better than anodal in treating OCD patients with an extracephalic montage [23, 33].

## Method/design

### Aim

#### Primary objective

The main aim of this feasibility trial will be to inform the development of a subsequent definitive tDCS full-scale trial. This feasibility trial will collect information about recruitment, study conduct, tDCS delivery, and assessment methods to inform future trial design.

The objectives of this feasibility trial will be to assess:

•Acceptability and safety of the intervention

•Feasibility of recruitment

•Adherence and tolerability of tDCS and study assessments

•Willingness of clinicians to recruit participants

•Practicality of applying tDCS in the clinical setting

#### Secondary objectives

These clinical outcomes will enable the project team to further refine the methodology to ensure optimal efficiency in terms of both delivering and assessing the treatment in a full-scale trial.

•Optimal stimulation target (OFC, SMA)

•Likely magnitude of effect of the intervention on OCD symptoms, to determine standard deviation of the outcome measure, to estimate sample size

•Duration of effect of stimulation, to space treatments efficiently

•Usefulness and limitations of specific neurocognitive tests to determine a definitive test battery

Additionally, qualitative data will be collected from participants to better understand their experience of taking part in a tDCS intervention, as well the impact on their overall quality of life.

### Design

This feasibility study is designed as a double-blinded, sham-controlled, crossover randomised multicentre design in adults with OCD.

### Ethical approval

REC approval was granted to this study on 29th March 2019 (REC ref: 19/EE/0046). This study is co-sponsored by the University of Hertfordshire and Hertfordshire Partnership Foundation NHS Trust.

### Participants

**Table:** 

Inclusion criteria	Exclusion criteria
• Community-based service users, aged 18 years or older. If the clinician has any concerns about the participant’s cognitive competence for the completion of the CANTAB assessment, a MoCA (Montreal Cognitive Assessment) will be conducted. Only those with no indication of cognitive impairment will be eligible. • DSM-5 defined obsessive–compulsive disorder determined by a research psychiatrist using the structured interview for DSM-5 • Duration of symptoms >1 year (from medical history) • Baseline score ≥ 20 on the Yale-Brown Obsessive–Compulsive Scale (Y-BOCS) • Ongoing medication (SSRI, tricyclic antidepressant, antipsychotic, benzodiazepine) is allowed as long as the dose is kept stable for a sustained period before (≥ 6 weeks) randomisation and remains so throughout the study. • CBT is not allowed during or within 6 weeks of the start of the intervention. • If patients have changed medication in the last 6 weeks or are receiving treatment already with CBT, they will need to return to the clinic after 6 weeks and then be randomised.	• History of psychotic disorder (schizophrenia, psychotic symptoms, bipolar disorder), Tourette syndrome (tic disorders not amounting to Tourette syndrome will not be exclusionary), organic mental disorder, psychosurgery, personality disorder of borderline or histrionic type • Alcohol/substance-abuse disorders within the past 12 months • Any other DSM-5 disorder that is considered the primary focus of treatment • Severe depression, defined by a Montgomery-Åsberg Depression Rating Scale (MÅDRS) score > 30 at baseline • Actively planning suicide (scoring > 4 on item 10 of MÅDRS) or judged by the clinician to be at significant risk of self-harm • Received CBT involving exposure response prevention (ERP) from an accredited (British Association of Behavioural and Cognitive Psychotherapies (BABCP) approved or equivalent) therapist (e.g. IAPTs stage 2) in the last 6 weeks • Highly CBT resistant (inadequate clinical response, equivalent to < 25% improvement); ≥ 3 previous adequate (> 12 weeks) trials of CBT involving ERP from an accredited (BABCP-approved or equivalent) therapist • Highly medication resistant (inadequate clinical response, equivalent to < 25% improvement); ≥ 3 previous adequate (> 12 weeks) trials of any SSRI or clomipramine taken at optimal doses with adequate adherence • Needing regular psychotropic drugs other than permitted medication. • Currently involved in a treatment research study • Acute or unstable physical illness • Skull defects, or skin lesions on scalp (cuts, abrasions, rash) at proposed electrode sites • History of surgical procedure with implanted body materials or devices (e.g. metal, pacemakers) • Epilepsy or other clinically defined neurological disorder or insult • Inadequate understanding of English to give informed consent or such that the outcome measurement is impossible • Women of child-bearing age not using reliable contraception (e.g. oral contraception pill, intrauterine contraceptive device or condom). • Pregnant or breast-feeding women

### Recruitment

**Fig. 1:**
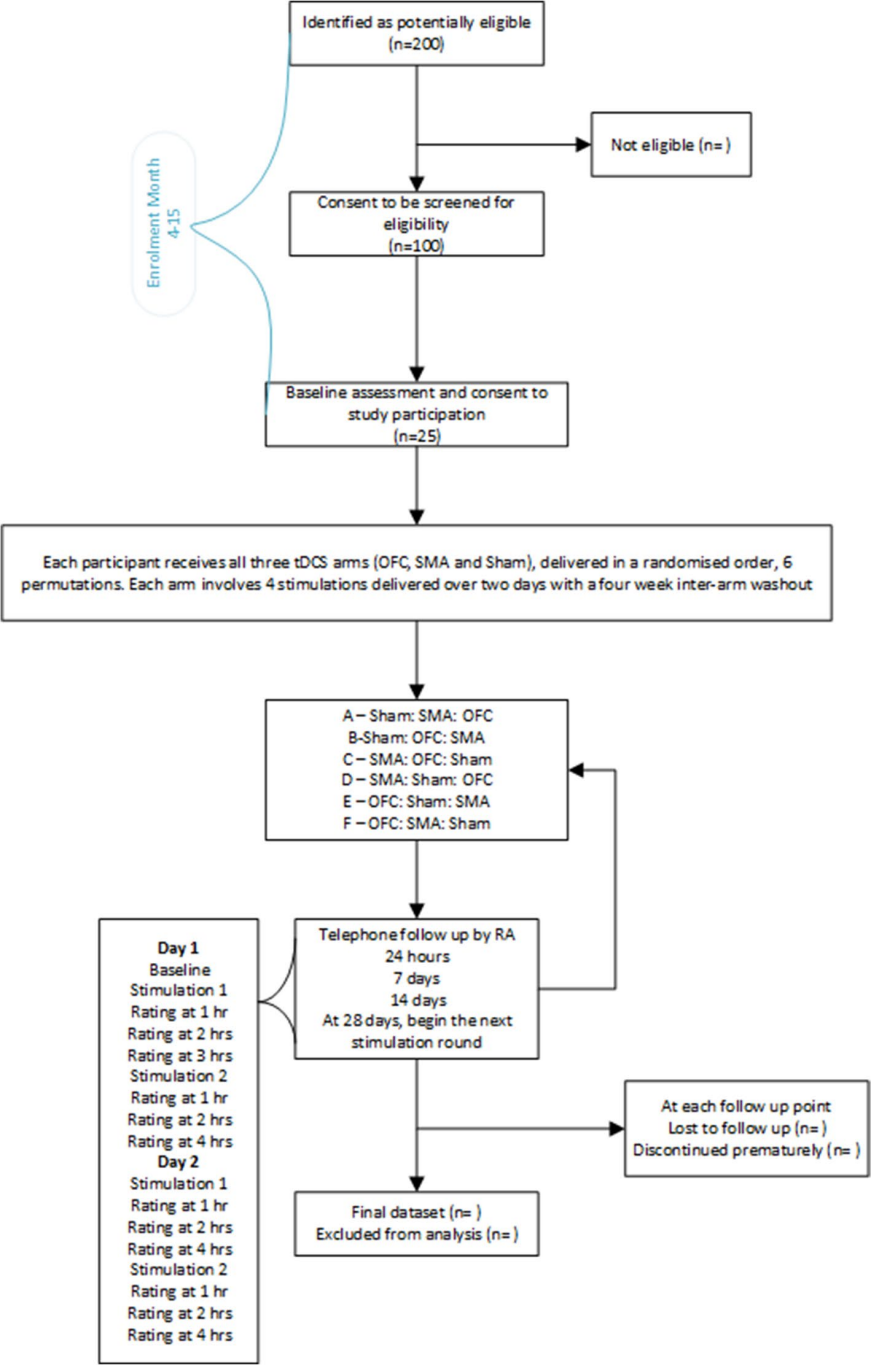
Trial flow chart

Recruitment will primarily take place at 2 outpatient centres: Rosanne House (Welwyn Garden City, Hertfordshire Partnership University NHS Foundation Trust) and University Department of Psychiatry, College Keep, Southampton. Camden and Islington NHS Trust will also act as a Participant Identification Centre (PIC). It is expected that 200 patients will be screened to allow for recruitment of 25 patients (Fig. 1). This will provide a robust estimate of the ascertainment ratio for recruitment to the study (limits within ± 10%).

Patients will be identified for screening from OCD clinics, primary health care services (e.g. Improved Access to Psychological Therapies (IAPTs)), charity/support networks, adverts/promotional material and Trust databases. Members of the research team will make regular contact with the relevant referring centres to ensure potential patients are referred. Trust databases will be screened for patients receiving routine care with a diagnosis of OCD. The research team will discuss their case with the treating clinician, and the clinician will refer the patient to the OCD service if deemed appropriate.

#### For patients referred from routine care to the OCD Service or the study

A member of the research team will identify adults with OCD who have been referred from routine care to the OCD Service (HPFT), or to the Mood and Anxiety Disorders Service (Southampton), or to the study, and will complete a screening log. The research team will either send the participant information sheet (PIS) about the trial or approach and provide this to the patient in the clinic and ask if they would be interested in taking part in the study. If agreeable, the patient will be sent a screening consent form and prepaid envelope. A clinician will be available to call the patient to discuss any queries that they may have. The patient will be asked to sign the consent form and post it to the research site; once received, the study clinician will arrange a convenient time to conduct the screening phone call. At the beginning of the screening phone call, the clinician will talk through the consent form with the patient to ensure that they have fully understood what they are consenting to, then countersign the signed consent form that had been sent by the patient.

#### For patients identified from primary health care services (e.g. IAPTs), charity/support networks, adverts/promotional material

OCD charity and support networks have given their support to the protocol and will advertise the study to their membership. Any patients who express an interest in the study will be sent information about the trial which will give a number to call to discuss further. Potential participants who respond to an advert or promotional material about the study will be sent information about the trial by one from the research team. Adverts/promotional material may be placed in primary health care services, health clinics attracting high levels of OCD patients, local newspapers, appropriate websites and relevant noticeboards. Research team members may also advertise the study through other media channels (e.g. local radio, patient support groups). Patients who express interest will be contacted by the researchers to discuss the study. If agreeable, a consent form and prepaid envelope will be sent. A clinician will be available to call the patient to discuss any queries that they may have. The patient will be asked to sign the consent form and post it to the research site, once received the clinician will arrange a convenient time to conduct the screening phone call. At the beginning of the screening phone call, the clinician will talk through the consent form with the patient to ensure that they have fully understood what they are consenting to, then countersign the signed consent form that had been sent by the patient.

#### For patients identified from the Trust databases

Patients who have a probable diagnosis of OCD on Trust databases will be identified by the clinical studies officers, research nurses, research assistants or research doctors. The treating clinician will be contacted and the suitability of the patient for the study discussed. If the treating clinician believes it is appropriate, the patient will then be referred to the study team for potential recruitment. Information about the trial will also be disseminated to clinicians. If they have suitable patients, they will refer them to the study team. The patient, if agreeable, will then be contacted by a member of the research team to see if they are interested in participating in the study. If interested, the patient will be sent an information sheet, consent form and prepaid envelope. A clinician will be available to call the patient to discuss any queries that they may have. The patient will be asked to sign the consent form and post it to the research site; once received, the clinician will arrange a convenient time to conduct the screening phone call. At the beginning of the screening phone call, the clinician will talk through the consent form with the patient to ensure that they have fully understood what they are consenting to, then countersign the signed consent form that had been sent by the patient.

### Screening

Patients will consent to be screened for the study. A member of the research team will assess eligibility according to the eligibility criteria and take a brief medical history. The results of all screening will be recorded both in the patients’ medical notes and on the study case report form (CRF). Eligibility criteria will be confirmed by the clinician who will be delivering the intervention.

### Randomisation scheme

Patients enrolled will be randomised to one of six permutations (4 per group):

- A—Sham: SMA: OFC
- B—Sham: OFC: SMA
- C—SMA: OFC: Sham
- D—SMA: Sham: OFC
- E—OFC: Sham: SMA
- F—OFC: SMA: Sham

The participant will sign the consent to study enrolment and randomisation. The clinician will have authorised access to the online randomisation programme. They will be provided with an identification code and password to access the randomisation programme. The clinician will enter the specific internet site, using their username and password and enter the study Patient Identification Number (PID), which includes the centre code. The computer will allocate the next consecutive randomisation allocation recorded automatically on the CRF. The clinician will record which intervention the patient was randomised to in the patients’ medical records and in a sealed envelope into the trial master file (TMF).

### Blinding

There will be two levels of blinding. The patient will be partially blinded as they will be able to tell which area is being stimulated (OFC/SMA), but they will not know whether it is sham/active stimulation as this will be preprogrammed. The RA doing the questionnaire outcome measures with patients will be fully blinded as they will not know the stimulation target or whether it is sham/active. In the event of an adverse event, the RA will report it to the clinician administering tDCS (unblinded) who will assess and manage it; patient unblinding will be considered if deemed appropriate.

### Medical device

Transcranial direct current stimulation (tDCS) devices will be used in this study. This product has been CE marked, approved for depression, chronic pain and post stroke rehabilitation; it will be used as off label within the OCD population. All manufacturer guidance will be adhered to when delivering the stimulation. These will be preprogrammed to deliver either an active current or a sham stimulation. The stimulator is a modular system for direct current (DC) stimulation, designed specifically for both research and clinical use. It consists of a stimulator (HDCstim), a programmer (HDCprog) and a set of electrodes (HDCel). The basic HDCkit package contains everything required to administer tDCS. The HDCkit is fully programmable and can run any number of applications or treatments within its parameters. This device is manufactured by Newronika (Italy) and distributed by Magstim (UK).

#### HDCprog

The HDCprog is an easy-to-use touch-screen LCD programmer that, when connected to a HDCstim, allows the clinician/researcher to set tDCS treatments or protocols for their patients or subjects (e.g. active stimulation or sham, polarity, current intensity and duration). By using the HDCprog, it is possible to define an entire treatment or application by setting the number of stimulations (maximum 99), the intensity (up to 1.5 mA per channel), the duration (maximum 20 min), and the minimum interval between two consecutive simulations (max 1168 h). Upon connecting it to a HDCStim after a treatment has been administered, the HDCprog allows the physician to check stimulation results through an easy-to-navigate stimulation report interface, allowing for an informed evaluation of applications administered so far. HDCprog is powered by an AC/DC adapter, which is certified for medical use.

#### HDCstim

The HDCstim is a battery supplied, programmable stimulator that is used to administer direct current (DC) stimulation. The HDCstim is only preprogrammable with a HDCprog and will not deviate from the treatment or protocol chosen by a researcher or clinician. The HDCstim delivers a finite number of prescribed stimulations with a selectable time interval between two consecutive stimulations. The HDCstim records all use and its outputs, for later retrieval on the HDCprog by researcher or clinician. It is lightweight, highly portable and compact. Additional HDCstims can be ordered as required without the need to buy additional programmers.

#### HDCel

The HDCel is a set of accessories for the administration of DC stimulation from the HDCStim. HDCel consists of a set of electrodes made from plant cellulose and conductive silicone, and are manufactured to ensure biocompatibility with the skin. Electrodes are safely connected to HDCStim through the cables provided, the connections of which are designed to minimise the risk of incorrect set-up. Personalised sponge electrode cover, standard EEG gel and saline solution 0.9% NaCl will be applied on the HDCel to ensure optimal electrical conduction.

### Intervention

#### Preparation and labelling of the medical device

The HDCprog will be connected to the different HDCstimulators in the set-up phase of the study, and for each HDCstimulator will be defined the entire treatment application as per protocol, by setting the type of stimulation (active or sham), the intensity (2 mA) and the duration (20 min). Stimulation will take place in a quiet room. The patient sits awake in a comfortable chair. Electrodes are placed using the International 10–20 System [39]. The settings on the tDCS stimulator, marked with “1” and “2” codes known to the clinician responsible for the tDCS montage and delivery, are preselected for active or sham stimulation [40]. The clinician responsible for the montage will be instructed regarding the target site (OFC or SMA) by the external randomisation allocation. Intervention settings are unknown to the patient and the RA thus tDCS is administered in double-blind methodology. Individual sponge electrodes soaked in saline solution and standard EEG gel will be used. The active electrode is placed over the SMA (cathodal, Fz point) or left OFC (cathodal, FP1 point) and attached to the scalp using a personalised sterile head net tubular bandage. The inactive (reference electrode) is placed over the right deltoid using an elastic band. In active treatment, a 2-mA current is delivered for 20 min. Sham stimulation uses the same methodology apart from the current setting on the stimulator. Previous studies indicate that this procedure is well tolerated by participants with mental disorders, and reliability of sham procedures [24, 27, 28, 40–43].

### Stimulation schedules

The stimulation protocol in FEATSOCS has been designed according to expert recommendations regarding the safety of tDCS, developed for research ethics committees and Institutional Review Boards, as well as in clinical practice [43]. The application of tDCS has presented minimal risk in numerous research or clinical studies when applied within standard parameters. Minimal risk means there have been no serious adverse events, that common adverse effects such as reddening of the skin are mild and short-lived, and that reasonable efforts at assessment have determined there is no evidence of brain damage. Standard parameters to date mean that (1) the current is less than 2.5 mA, (2) it is applied through electrodes that are known to minimize skin burns at the specific current level, (3) the current application duration is less than 20–60 min per session, and (4) sessions are not more frequent than twice per day [42, 43]. In line with these recommendations, each participant will receive two 20-min stimulations separated by 4 h on Day 1 and on Day 2 of each session comprising:

- Cathodal tDCS 2 mA to the bilateral SMA
- Cathodal tDCS 2 mA to the left OFC
- Sham tDCS to the OFC or SMA

The courses will be allocated in a randomised and counterbalanced order. Each 2-day course of tDCS will be spaced 4 weeks apart to avoid carry-over effects, and allow for the duration of effect to be evaluated. Patients will be assessed before each stimulation, up to 4 h afterward, with the assessments at 4 h after the second stimulation may be conducted over the phone, and then at 24 h and 7,14 and 28 days following tDCS stimulation.

### Measures

#### Baseline data

The following assessments will be used to gather baseline data (all outlined in Table 1).

- Yale-Brown Obsessive–Compulsive Scale (Y-BOCS) [44]

- Yale-Brown Obsessive–Compulsive Challenge Scale (Y-BOCCS Challenge): challenge version of the Y-BOCS (10), which is designed for researchers or patients to evaluate short-lived changes in symptom severity using a visual analogue scale [45]
- Clinical Global Impressions Scale (CGI-S) [46]
- Montgomery-Åsberg Depression Rating Scale (MÅDRS) [47]
- Barratt Impulsiveness Scale (BIS) [48]
- Sheehan Disability Scale (SDS) [49]
- Neurocognitive tests: CANTAB (Stop Signal Reaction Time Test, Intra-Extradimensional Set Shift), Prisoner’s Dilemma test (cooperation test). The Fabulous Fruit game (habit learning test): only patients who have attended a screening appointment in person prior to the 13th March 2020 (when research activity was paused due to COVID-19) will be assessed using the Fabulous Fruits Game on stimulation days. Patients who are screened over the phone, will not be familiarised with the Fabulous Fruits Game (as it is not possible to do this remotely) and therefore will not be assessed using the Fabulous Fruits Game on stimulation days.

**Table 1:** Table of study assessments

Conducted By	Test	Screening (completed by Clinician)	In centre assessments for each of 3 sessions (Sham, OFC, SMA) Day 1										In centre assessments for each of 3 sessions (Sham, OFC, SMA) Day 2									Follow-up via telephone^b^		
			Baseline	Intervention	1 h	2 h	3 h	4 h	Intervention	1 h	2 h	4 h	0 h	Intervention	1 h	2 h	4 h	Intervention	1 h	2 h	4 h	24 h	7 days	14 days
DR	DSM-5	X																						
DR/RA	Y-BOCS	X	X										X									X	X	X
DR/RA	Y-BOCS Challenge		X		X	X		X		X	X	X	X		X	X	X		X	X	X	X	X	X
RA	CGIS		X										X									X	X	X
DR/RA	MÅDRS	X	X																			X	X	X
RA	BIS		X																					
RA	SDS		X																			X	X	X
RA	CANTAB		X				X																	
RA ^a^	AEs		X		X	X		X		X	X	X			X	X	X		X	X	X	X	X	X
DR	Clinical Review		X					X			X		X				X			X				
	**Time (min)**	**60**	**105**	**20**	**15**	**15**	**30**	**30**	**20**	**15**	**15**	**30**	**30**	**20**	**15**	**15**	**20**	**20**	**15**	**15**	**30**	**15**	**15**	**15**

^a^The RA will complete the AE questionnaire with participants and refer to the clinician if necessary

^b^There will be an additional telephone call at 28 days only after the patients have completed the last arm of the intervention. The assessments will be the same as the previous phone calls (prior to this, day 28 will be day 1 of the next intervention arm that the patient completes)

Adverse events will be recorded using a questionnaire developed by Brunoni et al. [50] developed specially for tDCS. This asks patients to rate 10 symptoms on a scale of 1–4 (see Table 2).

**Table 2:** tDCS Adverse Effects Questionnaire

tDCS Adverse Effects Questionnaire – Session ____________________			
Do you experience any of the following symptoms or side effects?	Enter a value (1–4) in the space below (1, absent; 2, mild; 3, moderate; 4, severe)	If present: Is this related to tDCS? (1, none; 2, remote; 3, possible; 4, probable; 5, definite)	Notes
Headache Neck pain Scalp pain Tingling Itching Burning sensation Skin redness Sleepiness Trouble concentrating Acute mood change Others (specify)			

#### Trial assessments

•Yale-Brown Obsessive–Compulsive Scale (Y-BOCS)

•Yale-Brown Obsessive–Compulsive Challenge Scale (Y-BOCCS Challenge)

•Clinical Global Impressions Scale (CGIS)

•Neurocognitive tests: CANTAB (stop signal reaction time test, intra-extradimensional set shift), Prisoner’s Dilemma test (cooperation test), Fabulous Fruit game (habit learning test—only to be conducted with patients who have been screened for eligibility in person prior to COVID outbreak in March 2020, and familiarised with the process prior to attending the research site for stimulation).

•Adverse Events Questionnaire

#### Long-term follow-up assessments

Patients will be followed up 7, 14 (via telephone) and 28 days (in clinic at their next stimulation cycle and in clinic or via telephone at the last study follow-up planned 28 days after the final stimulation) after their participation in the study, using the Y-BOCS, Y-BOCCS challenge, MÅDRS, SDS and the CGIS by the RA. Any reported adverse events will be followed up by the clinician by phone; if deemed necessary, the patient may be asked to come back to the clinic.

#### Qualitative assessments

Patients will have the option to take part in one-to-one interviews conducted by the RA at two time points in the study: the first, prior to the first visit, and the second, once the patient has completed all three arms of the study. A semi-structured topic guide informed by existing literature will be developed following consultation with PPI representatives. This will ensure that elements of the lived experience and illness perceptions that are of importance to this patient group are captured by this research.

Should patients opt to discontinue the study at any point after providing consent, they will be offered an exit interview to discuss their experiences and motives for discontinuation with the RA.

All research staff (across both sites) will also be invited to be interviewed by the RA (based at HPFT) concerning their experience of working on the study and specifically their thoughts around the feasibility of a full-scale tDCS trial.

### Trial involvement

Patients will be asked not to alter their average intake of coffee and avoid alcohol the day before tDCS. They will be also asked not to drive after receiving the intervention; therefore, reasonable travel costs will be reimbursed if they have made relative arrangements or a taxi will be booked and paid for. This will be explained to potential participants in the information sheet. There is no financial reimbursement associated with the time commitment to the study.

#### First visit (Day 1)

•Patients will be consented then randomised by a clinician who will later deliver the intervention. As patients will be unable to drive after receiving the stimulation, the clinician will check that suitable transport arrangements are in place.

•Pregnancy test the first day (if applicable); pregnancy and reliable contraception questionnaire at each visit

•The RA will then complete baseline assessments with the patient, as in the table of study assessments (see Table 1).

•The patient will then be taken to a room where the intervention will be delivered by the clinician. Blood pressure and heart rate will be measured by the clinician before and after the stimulation.

•After the 20-min intervention is complete, the patient will be given somewhere to wait, and will be followed up by the RA on a regular basis using the Y-BOCS challenge as well as noting any adverse side effects. A questionnaire developed by Brunoni et al. (2011) designed to record adverse events (AEs) related to tDCS will be used.

•Neurocognitive tests at 3 h

•Four hours after the intervention, the clinician will return to review the patient then deliver the intervention a second time, again for a duration of 20 min.

•The patient will be asked to wait again in the centre, and followed up by the RA for the duration of 2 h using the Y-BOCS challenge, as well as noting any adverse events (AEs).

•The clinician will review the patient before they leave, inclusive of checking the patient’s blood pressure and heart rate.

•The RA will phone the patient to conduct the assessments at 4 h after the second stimulation.

•It is anticipated that the patient will be in the centre for 8 h on the first visit.

#### Second visit (Day 2)

•The clinician will review the patient on arrival; they will then complete assessments as per the table of assessments with an RA (see Table 1).

•The clinician will then deliver the 20-min intervention. Blood pressure and heart rate will be measured by the clinician before and after the stimulation.

•The RA will follow up the patient on a regular basis using the Y-BOCS challenge, as well as noting any AEs.

•Four hours after the intervention, the clinician will return to review the patient and deliver the intervention a second time, again for a duration of 20 min.

•The RA will follow up the patient on a regular basis using the Y-BOCS challenge, as well as noting any AEs.

•The clinician will review the patient before they leave, inclusive of checking the patient’s blood pressure and heart rate.

•The RA will phone the patient to conduct the assessments at 4 h after the second stimulation.

#### Follow-up via telephone

•The RA will follow up with patient via telephone, 24hours, 7 days and 14 days after the second visit. The last study follow up will be 28 days after the final stimulation.

After a 4 week wash-out period, this 2 day cycle will be repeated using a different intervention according to which of the 6 permutations the patient is randomised to receive. Patients will be required to keep their current psychiatric medication dose stable and not to initiate cognitive behaviour therapy during the 12 weeks of the study. This is to avoid any effect of change in treatment being recognised as a change resulting from the treatment provided in the study. Should these restrictions prove unacceptable or a change in treatment is required for a clinical reason the patient will be withdrawn from the study.

### Withdrawal criteria

Willingness to take part in the study will be confirmed with the patient at the beginning of each round of stimulation (i.e. on Day 1), the patient may withdraw from the study at any point. The research clinician will also check that the patient remains eligible for the study for the duration of the time that they are a participant. Should a patient become ineligible, they will be withdrawn from the study. Furthermore, if a patient reports any serious adverse experiences, they will be withdrawn from the study.

### End of trial

The end of the trial will be defined as the date that the last patient has their 28-day follow-up and clinical review from the third round of the intervention. In order to assess compliance in patients receiving tDCS, at each of the follow-up visits, the research psychiatrist will record in the CRF information on attendance at scheduled stimulation sessions, length of sessions and the completion of the study assessments. The research psychiatrist formally assesses the patient’s tolerability, clinical status and willingness to continue and records this in the CRF.

If a patient is not attending the stimulation sessions, the clinician will attempt to contact, ascertain the reason from the patient and record in the CRF. The patient will be encouraged to continue to attend the planned stimulation sessions; however, if the patient no longer wishes to receive tDCS, they will be asked if they will still continue to be assessed by the research assistant. If they do not wish to continue with tDCS, the withdrawal from study treatment form will be completed in the CRF.

At the end of the trial, the patient will be referred by the research psychiatrist to the relevant mental health services for further assessment and treatment.

### Sample size calculation

Study sample size is evaluated with respect to the primary outcome (difference in the challenge Y-BOCS between tDCS and Sham), given that the aim is to estimate the likely effect size of a future trial. We assume that the sample will be representative of the population, and that the observed variance, and Y-BOCS scores in each condition will allow for estimation of a typical group comparison. Given that there are only 4 exposures of tDCS in each study condition, with 4 weeks between exposures, we assume no carry-over effects, which will be tested using a mixed model. Each tDCS target (OFC and SMA) will be considered independently against Sham. Published estimates of effect size vary widely [45]; thus, a conservative estimate of *d* = .3 is assumed. Following Cocks and Torgersen [51], assuming *α* = 0.05, 1 – *β* = 0.80, a sample size of 20 per group allows detection of a lower limit of *d*’ > 0.1 for an expected effect size *d*’ = 0.3. An additional 5 patients will allow for a 25% dropout.

It is estimated that approximately 25 patients will be recruited from a pool of 200 patients identified from screening, giving power to estimate the ascertainment ratio to within ± 6%. The proportion of missed treatment sessions is expected to be less than 20% from a total of 206 sessions, giving a precision within ± 6%. At the outset of the study, it is not possible to estimate the likely number of reported adverse events (safety), which is expected to be low, and cannot therefore estimate the likely precision of estimation.

Time-dependent changes in the Y-BOCS scores, and in the CANTAB test scores will be considered using standard paired comparisons (*t* test). A sample size of *N* = 20 allows for detection of an effect size *d*’ = .65 with power 1 – *β* = .78, and an effect size *d*’ = .77 with power 1 – *β* = .90. A time-dependent model will be evaluated (mixed model) to examine trends over time, but with limited power.

### Statistical analysis

The study is designed to evaluate the feasibility of delivering a crossover randomised trial of the tDCS treatment intervention, with a limited sample size. The primary aims described above are focused on the feasibility of delivering the trial, and the associated analysis will be descriptive. The secondary analysis considers the clinical and neuropsychological outcomes, and will consider the potential effect size of the intervention, change in clinical effect over time, and the effect of the tDCS intervention on neuropsychological status.

The analysis will consider:

#### Acceptability, tolerability and safety

1. Treatment logs will be maintained, and the number of completed, shortened and missed sessions will be summarised by treatment condition. Reasons for refusal to complete or missing a treatment session will be recorded and tabulated. Comparison between treatment conditions will be evaluated using a McNemar test.
2. Incidents related to safety and tolerability will be logged during the exposure to tDCS and in the following weeks. The nature of the adverse events will be documented, classified (as outlined in the “Measures” section) and listed. The number of scope of the events in each of the study conditions will be compared using a McNemar test.

#### Feasibility of recruitment

The flow of patients through the study, from identification to randomisation, to completion of the study outcomes will be logged, allowing the ascertainment ratio to be estimated. The proportions of patients agreeing to be screened and randomised will be described. The reasons for refusal to take part will be documented and listed. It is expected that approximately 200 patients will be screened to recruit to target, such that proportions can be estimated to within ± 10%.

#### Adherence to tDCS and study assessments

1. Adherence will be evaluated by estimating the proportion, and range of sessions completed. The total number of sessions per patient will be 12, giving a limited power to evaluate differences between treatment conditions, but where comparison is possible, relevant analysis will be undertaken.
2. There are a range of study assessments that will be completed. The number of missing assessments or responses will be estimated for each patient, and comparison by treatment intervention will be evaluated using the McNemar test.
3. Reasons for non-completion will be recorded and listed by treatment intervention allowing comparison between the treatments.

#### Willingness of clinicians to recruit participants

The numbers of patients identified, screened and randomised will be evaluated by study site. Differences between sites will be considered and potential differences, such as the willingness of clinical staff to refer patients, considered.

#### Analysis of secondary outcome

##### Effect of tDCS

To evaluate the effect of tDCS on OCD symptoms, Y-BOCS scores at all time points in the 3 treatment conditions (Sham, OFC, SMA) will be estimated. Comparison of the OFC and SMA targets will be considered using paired tests to determine if one target is superior to the other. As the sample size is small, it is not expected that this study will have the power to evaluate with certainty the superiority of either target, but may provide an indication for one stimulation target over the other.

##### Likely effect size

The primary focus on comparisons will be on the potential effect size at 24 h after the final stimulation in each treatment condition. The observed and the lower limit for the effect size for the OFC and SMA targets will be evaluated with respect to the Sham condition, assuming that no carryover effects are observed in the crossover design (mixed model). A lower limit for effect size of < 0.1 will indicate a positive signal to proceed. Where possible, treatment target differences will be adjusted in a mixed model to account for baseline values of key patient variables.

##### Duration of effect

Change over time will be evaluated separately for the OFC and SMA targets using paired tests, identifying the time point (1, 2 and 4 h or 1, 7 and 14 days) at which the Y-BOCS returns to pre-stimulation levels. A mixed model may be evaluated where this may provide additional useful information about the trajectory of the Y-BOCS score over time (growth models).

##### Evaluation of neurocognitive test

The effects of the tDCS target on neurocognitive function will be evaluated for each target and test separately, by documenting test scores at each time point (baseline, 3 h). Differences in the profile of effect will be evaluated on the test dimensions dependent on the target (SMA target: motor impulsivity; OFC target: cognitive flexibility, cooperation). Comparison will proceed using paired tests, and if study power permits, mixed models.

### Progression to a full trial

The decision whether to progress will be based on consideration of all findings, but principally on the following data:

- The acceptability of tDCS will be considered throughout the ascertainment ratio (target 10%), willingness of clinicians to recruit to the study, and the numbers of patients citing tDCS as the reason for refusal (< 20%).
- Feasibility of recruiting sufficient patients in a multicentre national study will be evaluated in relation to the population size in each of the study centres, and extrapolation to the number of centres required to achieve the required sample size for a full-scale trial.
- The study targets a lower limit for the effect size of tDCS > .1, giving confidence that the effect of tDCS is at least .3. The study will enable evaluation of the superiority of the two tDCS target sites (OFC or SMA, given limited power).
- Differences between the targets will be considered in the light of differences in the profile of the neurocognitive tests indicating that the changes are consistent with the hypothesised effects (SMA target: motor impulsivity; OFC target: cognitive flexibility, cooperation).

### Trial monitoring and oversight

This trial was assessed to be low risk as the medical device being used is already CE marked and being used in other conditions such as depression and chronic pain. Therefore, in addition to the small sample size, it was not deemed necessary to have a separate data monitoring committee. Instead, any data monitoring reports alongside any safety concerns will be reported to and discussed by the study Trial Steering Committee (TSC). This is chaired by a professor with experience of using tDCS in depression, and comprised of the grant applicants, Public and Patient Involvement (PPI) representatives and an independent statistician.

The trial manager will be responsible for conducting monitoring visits at both sites, and will report any findings to an independent monitor who will follow up with the sites remotely. Any serious adverse events will be reported to the sponsor and the REC within 7 days, and any unexpected serious adverse events will also be reported to the manufacturer of the tDCS machines within 15 days.

### Data management

Each participant will be assigned a unique identifier when they attend either centre and consent to be screened for eligibility. All data will be recorded on an online database CRF (Castor) using the unique identifiers. The database will ensure that the blinded rater remains blinded. There will be a log at each site that denotes which members of staff are blinded. A copy of the rights allocated in Castor will also be stored in the electronic trial master file. Access to the database will be restricted and controlled to authorised personnel and will be password protected. Hard copies of consent forms will be stored securely at each site. All trial data will be kept for a minimum of 10 years. All study-related interactions will also be logged on local clinical systems where patient notes are routinely recorded. Data completeness will be assessed remotely by the independent data monitor and monitored routinely by the trial manager. Any missing data or deviations from the protocol will be file noted, to be considered when analysing the data.

### Public and patient involvement

The research question arose from the NIHR Obsessive–Compulsive and Related Disorders Clinical Research Group (OTOCARD-CRG), the aim of which was to advance OCD treatment through research. PPI representatives with lived experience of OCD, comprising one third of the CRG and including the Director of a leading OCD charity, participated fully in discussions with expert OCD researchers at teleconferences and a face-to-face research-planning meeting (Jan 2015). They judged the investigation of noninvasive neurostimulation to be of the utmost importance for patients and carers to advance treatment and an acceptable approach for people with OCD. They approved the principles of the design and were actively involved in designing the protocol. The PPI reference group for the current application each have lived experience of OCD. They include a director of an OCD charity (“Triumph Over Phobia” (TOP), a science journalist and author acting as a full team member (co-applicant) and a patient with prior experience of research governance for an OCD treatment trial. They have been actively consulted throughout the application process and supported the current application in the form of a feasibility study. They ensured the study methods are acceptable for patients, and the study information provided is accurate and comprehensible. They will remain involved in reviewing design, governance issues and supporting recruitment by raising awareness of the study among charity members. They will be integral to ensure dissemination of the results using the right language to the relevant communities.

In order to confirm the key aspects of the methodology is acceptable to patients, we devised a questionnaire to elicit a broad range of opinion from people with OCD. OCD Action and Triumph over Phobia (TOP), two leading UK consumer and advocacy charities for OCD, and the University Patients in Research group (PIRg) disseminated the questionnaire to their membership. The results confirmed that people with OCD judged that the design including the intervention and number of study visits is acceptable and not unduly onerous for patients.

## Discussion

So far, tDCS has been poorly studied in OCD, and the evidence about its therapeutic potential is limited. Preliminary results from the existing uncontrolled studies and three randomised controlled trials do point to a possible role of tDCS in the treatment of OCD, the most promising brain areas for electrode application appear OFC and pre-SMA/SMA [22, 23, 26–29, 35, 37, 38]. There nevertheless remains uncertainty about the optimal stimulation target, montage, frequency, magnitude and duration of effect, acceptability, tolerability and practicality of applying tDCS in the clinical setting. Further studies in this field are warranted [23, 26–28, 52]. As existing data are inadequate to support a full-scale trial, FEATSOCS addresses key research questions and knowledge gaps to enable the design of the most efficient, cost-effective study. We therefore propose a double-blind, sham-controlled, crossover feasibility study comparing tDCS of the two most promising sites, SMA and OFC (four stimulations per montage), in nontreatment-resistant patients. Neurocognitive tests will be applied to search for preliminary evidence supporting the hypothesis that tDCS of the SMA and OFC act by altering neurocognitive mechanisms of relevance to OCD, and to identify potential mechanisms as targets for treatment development. We test the hypothesis, preliminarily explored in healthy subjects, that modulation of the SMA may act by improving inhibitory control and of the OFC by improving cooperativeness and executive function [53, 54].

The proposed study will complement and extend beyond the three efficacy studies of tDCS in treatment-resistant OCD patients and one in drug-naïve OCD patients currently underway and recruiting which are using different tDCS stimulation protocols and study sample characteristics [55, 56]. In addition, one tDCS efficacy pilot study is completed, but the results are still unpublished, another trial appears on hold, and one study investigating tDCS in Pediatric OCD is not yet recruiting [53].

There has been an increasing interest in investigating tDCS also in combination with other OCD treatments. A recent randomised, double-blind, sham-controlled trial assessed the safety and efficacy of tDCS as adjunctive therapy with fluoxetine in parctipants with moderate–severe OCD (*n* = 60) [57]. In the study design, cases were randomly assigned in 1:1 ratio to receive either a 20-min period of stimulation with tDCS (2 mA, three times per week for 8 weeks, anode placed over the left dorsolateral prefrontal cortex, cathode over the right orbitofrontal cortex) and fluoxetine (experimental arm) or fluoxetine only (sham control arm). Results showed significant clinical improvement with no statistical differences were detected between experimental and control groups. The tDCS was well tolerated, and no major adverse events were reported [57]. A recent quasi-experimental, uncontrolled study, revealed clinical improvement in OCD with no statistically significant difference between the exposure response prevention (ERP) monotherapy and tDCS monotherapy groups (except for the quality of life variable); however, the improvement in the pharmacotherapy-ERP combination sub-group was superior than the pharmacotherapy-plus tDCS sub-group [58]. To date, one study applying tDCS with CBT is completed but the results are still unpublished, one RCT of simultaneous tDCS and exposure-based cognitive behavioural therapy (CBT) results active but not yet recruiting. At present, there are also three mechanistic open label studies underway and incomplete, one study investigating enhancement of therapeutic learning in OCD using tDCS, one examining arbitration between habitual and goal-directed behaviour using fMRI neuroimaging after tDCS in OCD patients, and one investigating electroencephalography (EEG) predictors of response to tDCS in OCD [56].

Among the protocol limitations, we acknowledge that having limited resources available for this feasibility study, more recent tDCS evolutions and personalised approaches (such as the use of high-definition tDCS to improve focalisation, the use of IRM mapping for personalizing the electrode placement) were not available.

### Trial status

This FEATSOCS trial is currently underway and recruiting in both centres. Recruitment of participants started on 23rd July 2019 in Hertfordshire and on 7th November 2019 in Southampton. Owing to delays in the study set-up and the impact of COVID-19, a recruitment extension has been asked and granted for the last patient to be enrolled on 30th April 2021. This protocol is based on Version 5 (15.06.2020).

## Data Availability

An anonymised dataset with data dictionary will be made available via the University of Hertfordshire Research Archive. Access to the data will be made contingent on citing this article and citing the main trial findings.

## Abbreviations

AE: Adverse event
AR: Adverse reaction
CANTAB: Cambridge Neuropsychological Test Automated Battery
CBT: Cognitive behavioural therapy
CI: Chief investigator
CRF: Case report form
CTU: Clinical trials unit
DBS: Deep brain stimulation
d-TMS: Deep transcranial magnetic stimulation
ERP: Exposure response prevention
EU: European Union
EudraCT: European Clinical Trials Database
FDA: Food and Drug Administration
HPFT: Hertfordshire Partnership University NHS Foundation Trust
IAPTs: Improved Access to Psychological Therapies
ISRCTN: International Standard Randomised Controlled Trials Number
mA: Milliampere
NHS R&D: National Health Service Research & Development
NICE: National Institute Clinical Excellence
NIHR: National Institute Health Research
OCD: Obsessive–compulsive disorder
OFC: Orbitofrontal cortex
OTOCARD-CRG: Obsessive–Compulsive and Related Disorders Clinical Research Group
PI: Principal investigator
PIRg: University Patients in Research group
PIC: Participant Identification Centre
PIS: Participant information sheet
QoL: Quality of life
RA: Research assistant
RCT: Randomised controlled trial
REC: Research ethics committee
rTMS: Repetitive transcranial magnetic stimulation
SAE: Serious adverse event
SMA: Supplementary motor area
tDCS: Transcranial direct current stimulation
TMF: Trial master file
TMG: Trial Management Group
TOP: Triumph Over Phobia
TSC: Trial Steering Committee
UH: University of Hertfordshire
